# Diseases induced by malfuction of the enteromammary circle

**DOI:** 10.1186/1939-4551-8-S1-A273

**Published:** 2015-04-08

**Authors:** Isaac Azevedo Tenorio, Aderbal Sabra, Selma Sabra

**Affiliations:** 1 Unigranrio, Brazil

## Objectives and study

Breast milk is produced by the mammary gland under the influence of the enteromammary circle of the mother. The bipolar extremes of this circle is represented by the GALT system, in the GI tract of the mother. The other extreme is represented by the mammary gland.

The present study are developed to explore the broad expectrum of the clinical picture of children presenting symptoms exclusively breastfed.

## Methods

From January 2009 to January 2011, charts from 24 children, age 0 to 6 months, diagnosed with breast milk enteropathy where selected. All children where exclusively breast fed since birth.

## Results

The symptomatology related to the GALT, was blood in stools present 58,3% of the patients. Gastro-oesofageal reflux, was present in 41% of the patients, abdominal pain in 33.3%, diarrhea in 20.5% , constipation in16.6% , bulky stools, flatus and vomiting in 12.5%, colics and nauseas in 8,3% and hiccups in 4,1%.

In the BALT system, the respiratory tract show snoring and rhinitis present in 16,6% of the patients, followed by sinusitis and excess of catarrh with 8,3% and asthma and chronic couth with 4,1%.

In the SALT system, the skin show as the most frequent alteration the atopic eczema with a prevalence of 16.6%, followed by eczema of folds in 12,5%, pallor, erythema of the cheeks, perioral erythema and seborrheic dermatitis where present in 8,3% of the cases.

In the genetic background the family history of allergy show rhinitis present in both parents, in 37.4% of the father and 8,3% of the mother, intolerance to food in 16.6% of the mothers versus 8,3% of fathers. Asthma was present in 25% of the mothers versus 20,4% in the fathers.

## Conclusion

The present study design to explore the broad expectrum of the clinical picture of children exclusively breastfed reveal that the spectrum of the desease.

